# Citizen scientists’ engagement in flood risk-related data collection: a case study in Bui River Basin, Vietnam

**DOI:** 10.1007/s10661-024-12419-2

**Published:** 2024-02-17

**Authors:** Huan N. Tran, Martine Rutten, Rajaram Prajapati, Ha T. Tran, Sudeep Duwal, Dung T. Nguyen, Jeffrey C. Davids, Konrad Miegel

**Affiliations:** 1 Faculty of Agricultural and Environmental Sciences, University of Rostock, Rostock, Germany; 2 Faculty of Water Resources, Hanoi University of Natural Resources and Environment, Hanoi, Vietnam; 3 The Hague, Netherlands; 4 SmartPhones4Water, Chico, USA; 5 College of Land Management and Rural Development, Viet Nam National University of Forestry, Hanoi, Vietnam; 6 Smartphones For Water Nepal, Lalitpur, Nepal; 7 Faculty of Economics and Management, Thuyloi University, Hanoi, Vietnam; 8 Davids Engineering, Chico, USA; 9 California State University, Chico, USA

**Keywords:** Citizen science, Information and communication technology, Flood vulnerability, Land use, Low-cost rain gauge, Residential flooding

## Abstract

Time constraints, financial limitations, and inadequate tools restrict the flood data collection in undeveloped countries, especially in the Asian and African regions. Engaging citizens in data collection and contribution has the potential to overcome these challenges. This research demonstrates the applicability of citizen science for gathering flood risk-related data on residential flooding, land use information, and flood damage to paddy fields for the Bui River Basin in Vietnam. Locals living in or around flood-affected areas participated in data collection campaigns as citizen scientists using self-investigation or investigation with a data collection app, a web form, and paper forms. We developed a community-based rainfall monitoring network in the study area using low-cost rain gauges to draw locals’ attention to the citizen science program. Fifty-nine participants contributed 594 completed questionnaires and measurements for four investigated subjects in the first year of implementation. Five citizen scientists were active participants and contributed more than 50 completed questionnaires or measurements, while nearly 50% of citizen scientists participated only one time. We compared the flood risk-related data obtained from citizen scientists with other independent data sources and found that the agreement between the two datasets on flooding points, land use classification, and the flood damage rate to paddy fields was acceptable (overall agreement above 73%). Rainfall monitoring activities encouraged the participants to proactively update data on flood events and land use situations during the data collection campaign. The study’s outcomes demonstrate that citizen science can help to fill the gap in flood data in data-scarce areas.

**Supplementary Information:**

The online version contains supplementary material available at 10.1007/s10661-024-12419-2.

## Introduction

Globally, flooding impacts 100 million people each year and causes great economic losses (Jongman et al., 2015). To mitigate flood-related impacts, flood risk assessment is a pivotal task because it quantifies potential hazards and vulnerabilities associated with flood events to determine appropriate measures (de Moel et al., 2015). This task requires enormous amounts of data on flood hazards, land use information, and flood vulnerability (Apel et al., 2009). Unfortunately, flood data collection is hindered in undeveloped countries, particularly in Asian and African regions, due to time constraints, financial limitations, and inadequate tools (Glas et al., 2020; Huizinga et al., 2017; Sy et al., 2019). Typically, flood risk-related data are obtained from ground observations, hydrological and hydraulic modeling, remote sensing, and field surveys (Sy et al., 2019).

Traditional approaches, such as modeling and remote sensing in flood monitoring and flood risk assessment, have significantly advanced our understanding of these complex phenomena (Trinh & Molkenthin, 2021). These methods commonly document flooding and estimate flood impact for large areas under different scenarios or periods (Ferri et al., 2020). Modeling, which demands detailed data, provides accurate results (Apel et al., 2009). Advancements in information and communication technology (ICT), like cloud computing platforms, enable the rapid execution and analysis of analysis-ready satellite images. This facilitates the real-time or near-real-time display of flooding maps, providing valuable support to flood control operators for enhanced and efficient management (DeVriesa et al., 2020; Liu et al., 2018). However, modeling may be subject to uncertainties due to assumptions and limitations in input data and a lack of hydrological and hydraulic process understanding (Merz et al., 2010a, 2010b). Additionally, remote sensing can encounter challenges related to cloud cover, spatial resolution, and insufficient validation datasets (Schnebele & Cervone, 2013).

To address the mentioned limitations, various initiatives have been undertaken to incorporate citizen science in gathering data from past flood events (Sy et al., 2020), monitoring current flood situations (Ferri et al., 2020), and enhancing flood modeling (Azizi et al., 2023). Citizen science involves public participation in scientific research (Buytaert et al., 2014; Shirk et al., 2012) and is further facilitated by ICT that simplifies the collection of massive amounts of information and data (Buytaert et al., 2014). Data collected from communities through citizen science can be cost-effective (Buytaert et al., 2014), more spatially distributed, and relatively accurate (de Bruijn et al., 2019; Zeng et al., 2020). Therefore, citizen science is a promising approach for providing supplementary data to assess and manage flood risk (Scaini et al., 2021), allowing for on-the-ground observations and local insights to enhance the accuracy and completeness of flood-related data. In addition, citizen science projects may raise locals’ awareness of flood disaster prevention and build community resilience, thereby serving as a nonstructural measure in flood risk management (Ferri et al., 2020; Pandeya et al., 2021).

Public involvement in the collection of flood risk-related data on flood hazard, land use, and flood vulnerability has been discussed for the last two decades (Peters-Guarin, 2008; See, 2019; Sy, 2019). Citizen science has been widely applied in flood hazard assessment to determine the flooding extent (Sy et al., 2020), depth (Fohringer et al., 2015), flow velocity (Le Coz et al., 2016), and duration (Sy, 2019). Moreover, citizen scientists have contributed land use information through field data collection campaigns (Assumpcao et al., 2019) and online crowdsourcing platforms (Sparks et al., 2015). Finally, with regard to flood vulnerability, citizens have shared information about flood damage and their perspective on disaster management through field surveys conducted by researchers (Perera et al., 2015; Peters-Guarin, 2008). However, previous citizen science projects have rarely examined the power of locals’ data collection and contribution for these three mentioned data types in one citizen science program. In addition, some researchers have used citizen scientists to collect data without data validation or comparison with other sources (Assumpcao et al., 2019; Le Coz et al., 2016), which is necessary to understand the variations and limitations of this approach. Furthermore, many citizen science projects have collected data only once (Assumpcao et al., 2019; Perera et al., 2015) and have not utilized citizen scientists to monitor different floods, detect land use change (Tsiakos et al., 2019), and update flood damage (Merz et al., 2010a, 2010b).

The support of low-cost monitoring equipment and ICT paves the way for citizen science-based hydrological monitoring networks (Buytaert et al., 2014; Davids et al., 2019). For example, low-cost rain gauges or water level sensors installed in residential areas or public areas enable citizens to proactively and regularly monitor rainfalls (Davids et al., 2019; Fehri et al., 2020) and water levels (Pandeya et al., 2021; Weeser et al., 2018). These data can be transmitted wirelessly to a server or web-based platform (Pandeya et al., 2021) to provide up-to-date information for authorities and citizens. Data collection apps can gather the date and geolocation of measurements or surveys and take photos to enhance users’ understanding of the investigated objects (Davids et al., 2019). Furthermore, communication technologies can help scientists communicate with participants easily through social networks (Sy, 2019) to motivate them and retain their participation.

Vietnam is one of the Asian-Pacific countries that is most affected by natural disasters, particularly flooding (World Bank Group and Asian Development Bank, 2020). Floods are responsible for 97% of the total disaster loss (World Bank Group and Asian Development Bank, 2020). According to the World Resources Institute’s AQUEDUCT Global Flood Analyzer, as of 2010, river floods with a 10-year return period affected 2.4 million people and caused the gross domestic product damage of 6.0 billion USD (World Resources Institute, 2018). In addition, agricultural activities are mainly located in low-lying deltas and coastal areas, which attract more than 40% of the nation’s workforce (Ministry of Natural Resources and Environment, 2020). They are significantly affected by flooding and climate change (World Bank Group and Asian Development Bank, 2020). Although floods cause great losses, flood risk-related data to estimate potential flood damage remain inadequate in Vietnam (Chinh et al., 2016). Furthermore, the lack of locals’ involvement in flood risk planning and management (Dang et al., 2011; Pham, 2011) coupled with challenges in local-to-central collaboration (Garschagen, 2016) have hindered the effectiveness of flood mitigation measures. Therefore, it is necessary to find an integrated approach to gather missing flood data and enhance communication between locals and authorities to manage floods.

Our research aims to utilize the citizen science approach to collect flood risk-related data for the Bui River Basin in Vietnam, where citizen science-based studies are very limited. We recruited and trained participants living in or around flood-affected areas to self-investigate or investigate flooding in residential areas, land use information, and flood damage to paddy fields for 1 year. We compared the data obtained by citizens with those collected by the research team or the local authority to evaluate the quality of the citizen science data. In addition, we utilized a community-based rainfall monitoring network to engage participants in updating flood data during a data collection campaign.

## Study area

The Bui River Basin is located in northern Vietnam and is drained by two main rivers, the Tich and Bui Rivers, which flow through Hoa Binh and Hanoi provinces (

**Fig. 1:**
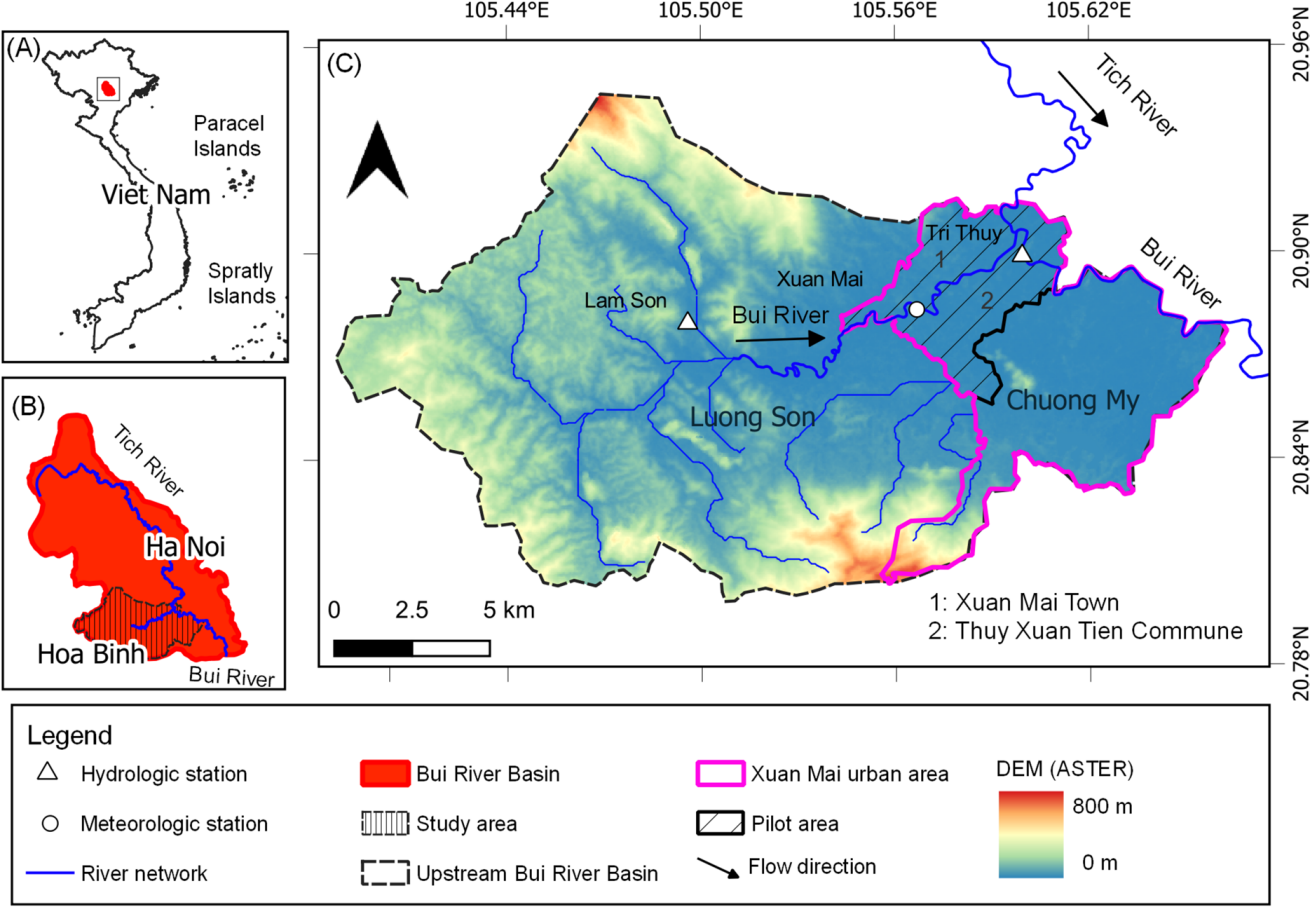
Location of the study area and pilot area: **A** the boundary of Vietnam and the location of the Bui River Basin; **B** the boundary of the study area and the two main rivers of the Bui River Basin; and **C** the river network system of the study area, the Xuan Mai urban area, and pilot area

Figure 1A, B). The study area, which spans 266.5 km^2^, is bounded by the upstream Bui River Basin originating from Lam Son hydrologic station area, Luong Son District, Hoa Binh Province, and the Xuan Mai urban area (

Figure 1C). The study area is characterized by semi-mountainous and semi-plain landforms (Doan & Bui, 2016) with elevations ranging from 0 m in the eastern region to 800 m in the northern and southern regions. The annual rainfall is approximately 1700 mm (Kieu et al., 2019), of which nearly 80% occurs in the rainy season from May to October.

The Xuan Mai urban area is known for the “Flooded Villages” of Hanoi located near the Tich-Bui Rivers conjunction area, 30 km from downtown Hanoi. According to Tran et al. (2022), the area has experienced more frequent and intense flooding over the last 15 years. The Tri Thuy station has recorded numerous high floods, including notable events in 2008, 2017, and 2018 (Tran et al., 2022). The 2018 historic flood, which had a 50-year return period (Tran et al., 2022), had an extreme effect on people, property, and agricultural production which is one of the main economic activities in the Xuan Mai urban area (Doan & Bui, 2016). Flooding in this area is caused by several factors, including intense rainfall leading to fluvial floods that overflow onto low-lying areas (Nguyen et al., 2019), mountain floods that flow directly onto low-lying areas (Le et al., 2022), and rapid land use change (Doan & Bui, 2016). Furthermore, the right bank area of the Tich–Bui Rivers serves as a flood retention area to reduce flood damage in downtown Hanoi (Hanoi People's Committee, 2009). This task poses challenges to agricultural production (Tran et al., 2021b). For example, in 2018, paddy-cultivated areas were inundated more than four times (Phan et al., 2019).

The Thuy Xuan Tien commune and Xuan Mai town in Chuong My District, Hanoi City, along the two sides of the Tich and Bui Rivers were chosen as pilot areas to investigate the applicability of citizen science for collecting flood risk-related data (Fig. 1C). The pilot area has Xuan Mai meteorologic station and Tri Thuy hydrologic station that have been measured daily since the 1960s. Furthermore, several academic institutes, such as schools and universities, are based in the area, creating a conducive setting for citizen science initiative implementation. School and university students belong to the most efficient forces in citizen science programs regarding the acquisition of knowledge and the use of data collection applications (Davids et al., 2019; Prajapati et al., 2021).

## Materials and methods

**Fig. 2:**
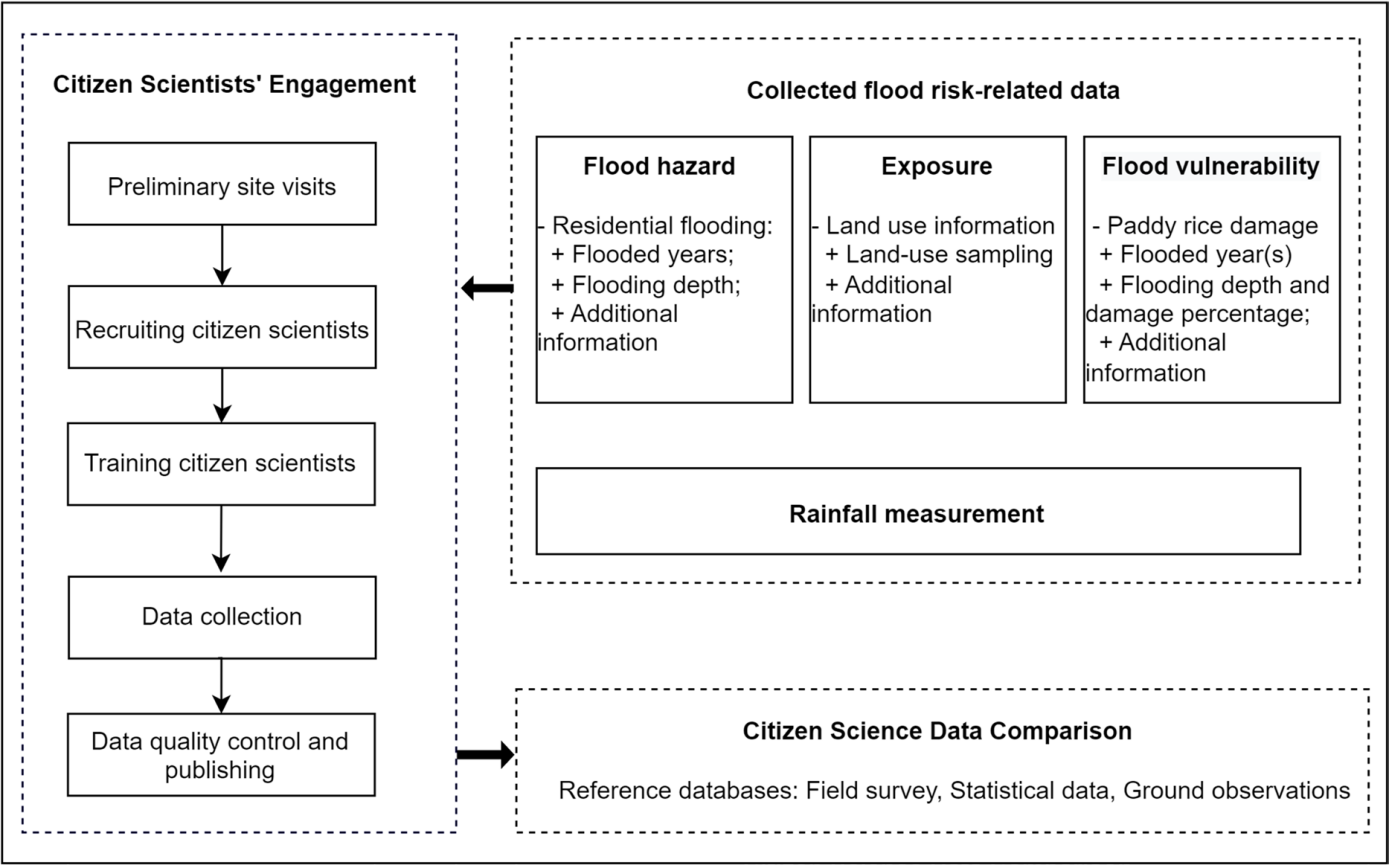
The research approach of citizen scientists’ engagement in flood risk-related data collection (Bonney et al., 2009)

To develop a community-based flood data collection approach, we implemented a citizen science program from September 2021 to August 2022. We followed the general approach suggested by Bonney et al. (2009), which consists of three main components: determining collected flood risk-related data, engaging citizen scientists, and comparing citizen science data (Fig. 2). These components are elaborated upon in the subsequent subsections. Our research developed a community-based rainfall monitoring network to promote a citizen science program and encourage participants to update flood risk-related data proactively. The reliability of rainfall collected by citizen scientists is beyond the scope of the current work and will need to be discussed in future research.

### Collected flood risk-related data

Flood risk assessment requires the collection of data on flood hazards, exposure, and flood vulnerability (Apel et al., 2009). For flood hazards, information on flood probability and intensity, such as flood extent, depth, and velocity data, is mainly addressed (Trinh & Molkenthin, 2021). For exposure, the land use map, building dataset, and population distribution are often used (de Moel et al., 2015). For flood vulnerability, flood damage functions that indicate the relationship between flood direct and indirect damage to objects (buildings, crops, people, etc.) are often considered (Merz et al., 2010a, 2010b). This study used a citizen science approach to collect data on the flooding depth in residential areas, the direct impact of floods on paddy fields (flooding depth and yield reduction) in the last 10 years, and current land use data in a pilot area. Information on the land use in the field was gathered and categorized into seven different classes: forest, shrubland, agriculture rice, agriculture non-rice, built low, built high, and water body. In addition, rainfall was measured for the whole study area using low-cost rain gauges as proposed by Davids et al. (2019). The low-cost rain gauge is described in Supplementary Material S1.

The questionnaire was designed to collect necessary data and included two parts. The first part covered the biodata questions of the respondents, and the second part covered the collected flood risk-related data. The questions about flood hazard and flood vulnerability data were based on the approach described by Glas et al. (2018). The question of flooding depth in the residential area was given using the reference height level of human body parts and houses to create easy-to-understand questions for citizen scientists (Peters-Guarin, 2008; Sy et al., 2020). The questions about exposure and rainfall data collection were adopted from Davids et al.’s work (2018, 2019), in which taking photographs of investigated subjects was obligatory. The questionnaire was designed on the Open Data Kit (ODK) Collect app and KoBo Toolbox web form for Android-based mobile devices and non-Android-based mobile devices, respectively, and paper forms (only applied for hazard and vulnerability data). The questions used to collect data in this research on the web form can be found in this link (https://bit.ly/3E5oNvX).

### Citizen scientists’ engagement

#### Preliminary site visits

Preliminary site visits were conducted in the Bui River Basin to understand the flood situation, choose the pilot area, and create reference datasets for comparison with citizen science data. During our site visits, ODK Collect was used to document flood marks left in residential areas and land use in the pilot area. The flood depth at 89 flood mark locations of the 2018, the biggest flood in the last 10 years, was measured using a tapeline (refer to Supplementary Material S2, Fig. S2). The measured flood depths depend on the clarity and accuracy of flood marks on buildings and other objects. Therefore, it is not guaranteed that these recorded flooding depths accurately represent the maximum water level of an actual event. Nonetheless, they do provide valuable information for reconstituting flood events. Land use data were collected at 14 sites where we could determine a typical land use class from seven classes within a 20-m radius. The land use class at sites was classified by the first author and controlled by the fourth author to ensure a consistent reference database (Saralioglu & Gungor, 2019). In addition, we gathered a 2018 flood report mentioning flood-affected areas and damage data on the agricultural production of individual farmers from the Chuong My District People’s Committee.

#### Citizen scientist recruiting and training

Citizens living in or around the pilot area who were over 12 years old, regardless of educational background, were the target group. Citizen scientists were recruited through personal relationships, social media, outreach at educational institutes, and field visits (Davids et al., 2019). Outreach was held for secondary school, high school, and university students, and the outreach program content was modified to match the participants’ backgrounds. The recruitment campaign occurred during the COVID pandemic, so seven outreach events were organized on site (*n* = 3), virtually (*n* = 1), or through hybrid meetings (*n* = 3). Citizen scientists interested in rainfall monitoring were equipped with low-cost rain gauges that were installed in their households. The citizen scientists were trained to conduct surveys or self-report data on their preferred questionnaire forms through in-class, virtual, or on-site training. To consolidate the training process, tutorial videos for the installation of data collection applications and the surveying procedures were published on the YouTube channel (https://bit.ly/44lfjHL; Vietnamese language only), and an annotated and added-picture demonstration was available on digital forms to guide the participants.

#### Data collection

The citizen scientists were categorized into two groups. The first comprised participants who self-reported or interviewed their family members on flood risk-related data or measured rainfall. These participants were called “self-investigators.” The self-investigators were asked to provide flood risk-related data at a feasible time within 2 weeks after the training session. After 2 weeks, the research team contacted the self-investigators again to thank them for their support, collect the completed paper forms, or invite them to provide data again. In addition, they were asked to monitor rainfall at their houses often during rainy days and less frequently on days without rain.

The second group comprised participants who participated in surveys to gather flood risk-related data from the locals. These participants were called “investigators.” The investigators were asked to conduct surveys using digital forms (ODK Collect, web form) after participating in training sessions. Each investigator was led to a specific area to conduct household surveys on flooding in residential areas and flood damage to paddy fields. Both investigators and self-investigators sampled land use in the field wherever they wanted during their daily life activities or data collection campaigns. To compare the results between citizen scientists and authors in land use classification, eight investigators participated in a field experiment for 1 day in April 2022. They were led to the 14 sites mentioned in the “Preliminary site visits” section to sample and classify land use.

#### Data quality control and dissemination

To enhance the citizen science data quality, completed questionnaires and measurements of flood risk-related data and rainfall data were reviewed manually in 2-week to 4-week intervals. Common errors included incorrect rainfall units, blurred images, and inconsistent data between answers or information and images. Feedback on errors was promptly provided to the citizen scientists to prevent implausible data. Mislocated coordinates caused GPS signal errors, and non-GPS-generated paper forms were processed based on the address of the survey areas, Google Maps, and survey photos (Beza et al., 2018; Ribeiro et al., 2020). All discrepancies were corrected, and edits and notes were documented for future analysis. Data collected through ODK Collect are publicly available on the S4W data collection platform on the website (https://data.smartphones4water.org/, retrieved on June 26, 2023) with the land use data category under development.

### Citizen science data analysis

To evaluate the reliability of citizen science data, we compared these data to the reference datasets created by authors or gathered by the local authority. Based on achievable reference datasets from preliminary site visits, the data of flooded and non-flooded points, and the paddy field flood damage rate in 2018, and land use samples gathered during the field experiment in April 2022 obtained from citizen scientists were compared. The comparison involved overall agreement (OA) and individual agreement levels, which were determined using a confusion matrix (Congalton, 1991). Rainfall comparison was excluded from the research.

For the flood hazard data, we compared flooded and non-flooded points gathered by citizen scientists with the flooding map for the 2018 flood. In addition, flood depth differences between citizen scientists and the flooding map at flooded points were tested. Following Ribeiro et al.’s approach (Ribeiro et al., 2020), a flooding map was created using a 1:2000 topographic map and the 89 flood depth points. The flood elevation of these points was determined by combining the flooding depth and elevation value. A local combination method was used to determine a typical flood elevation surface based on flood level points for each subdomain of 1 km × 1 km for a pilot area (Mason et al., 2021). A digital elevation model and flood elevation surface with 10 m × 10 m resolution for the whole area were created by performing the multilevel B-spline interpolation method in QGIS between elevation points and flood surface levels of distinct subdomains (Ribeiro et al., 2020). Pixels with elevation values lower than the flood surface level were flooded. The flooding map was validated using local authority reports, internet news, and permanent water bodies (Giordan et al., 2018).

For flood vulnerability, we compared the flood damage to paddy fields gathered by citizen scientists with official flood damage data from the local authority for the 2018 flood. The local authority only investigated damage information from households with damaged areas from 30 to 70% and more than 70% because this information is used for compensation claims. The questions about the paddy field damage rate in our research were classified in more detail with 20% damage intervals (i.e., 20–40%, 40–60%). Therefore, the paddy field damage rate obtained from citizen scientists used a median value (e.g., 30%, 50%) to compare with official data. The damage rate was reclassified to < 30%, from 30% to less than 70%, and ≥ 70%, corresponding to low, medium, and high levels, respectively. The flood damage collected by citizen scientists was acceptable when the damage rates matched the damage rate level.

## Results

### Citizen science data

#### Participant demographics

The participant demographics in this research are illustrated in Table 1 (for details on the participants, see Supplementary Material S3). There were 59 citizen scientists divided into two common genders. Most participants were 12–34 years old, accounting for 87%. In addition, 57% of participants were educated at the college level or lower, followed by 36% at the bachelor’s level. Forty-five percent of the participants were recruited through personal relationships, whereas only 2% joined the citizen science program through social media. The number of self-investigators was three times higher than the number of investigators.

**Table 1:** Demographic characteristics of citizen scientists

Variable	Categories	Number	Percent (%)
Gender	Female	30	51%
	Male	29	49%
Age^1^	12–17	22	39%
	18–33	27	48%
	34–60	7	13%
	> 60	0	0%
Educational background^1,2^	< Bachelor	33	57%
	Bachelor	21	36%
	> Bachelor	4	7%
Recruitment method	Outreach	13	22%
	Personal relative	27	46%
	Social media	1	2%
	Random visit	18	31%
Type of survey	Self-investigators	45	76%
	Investigators	14	24%

^1^Some participants did not provide this information. Therefore, not all categories total exactly 59 samples

^2^ < Bachelor’s, Bachelor’s, and > Bachelor’s indicate the highest education degree acquired or currently matriculated by the participants

#### Received data

**Fig. 3:**
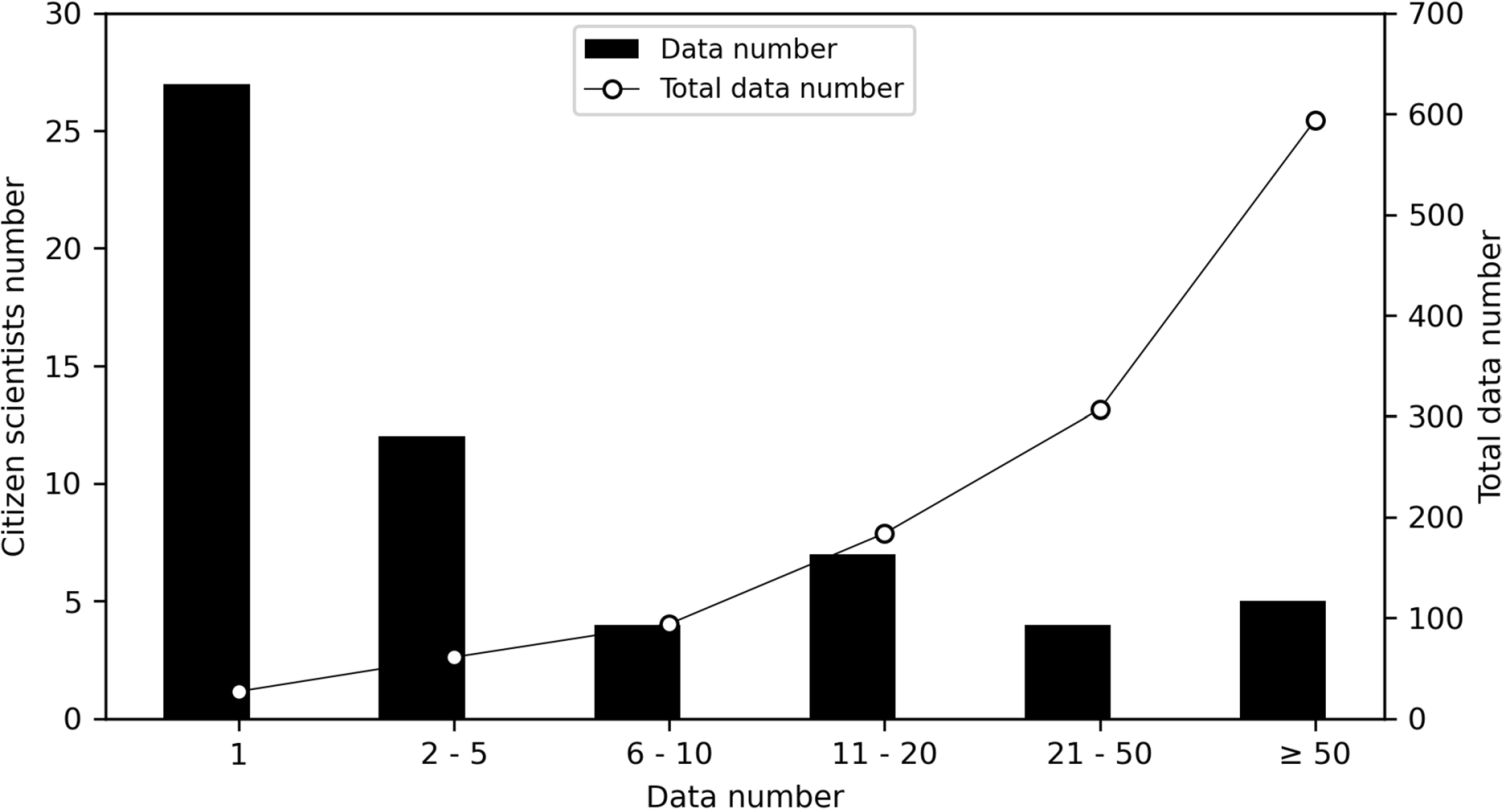
The number of citizen scientists and their data number

Fifty-nine participants contributed 594 flood risk-related data and rainfall measurements (hereafter referred to as data) for 1 year (Fig. 3). The allocation of the data number per participant decreased with the expanded data number per participant. Twenty-seven people, approximately 50% of participants, provided data only once. Only five participants, or 8.5% of citizen scientists, contributed more than 50 data per person. The citizen scientists who contributed more than 50 data during project periods were considered “active participants.” This active group contributed nearly 50% of the data over 1 year, increasing the total data number from 307 to 594.

The 594 data classified into four data types are shown in Table 2. The rainfall and exposure data accounted for 59% and 23% of the total collected data, respectively, which was significantly greater than the data for flood hazard and flood vulnerability (10% and 8%, respectively). The lists of measurements and surveys from citizen scientists are provided in Supplementary Material S4.

**Table 2:** The list of data categorized into data types

Type of data	Rainfall	Hazard	Exposure	Vulnerability	Total data
Number	348	62	138	46	594
Percent (%)	59%	10%	23%	8%	

### Data quality assessment

#### Flood hazard

**Fig. 4:**
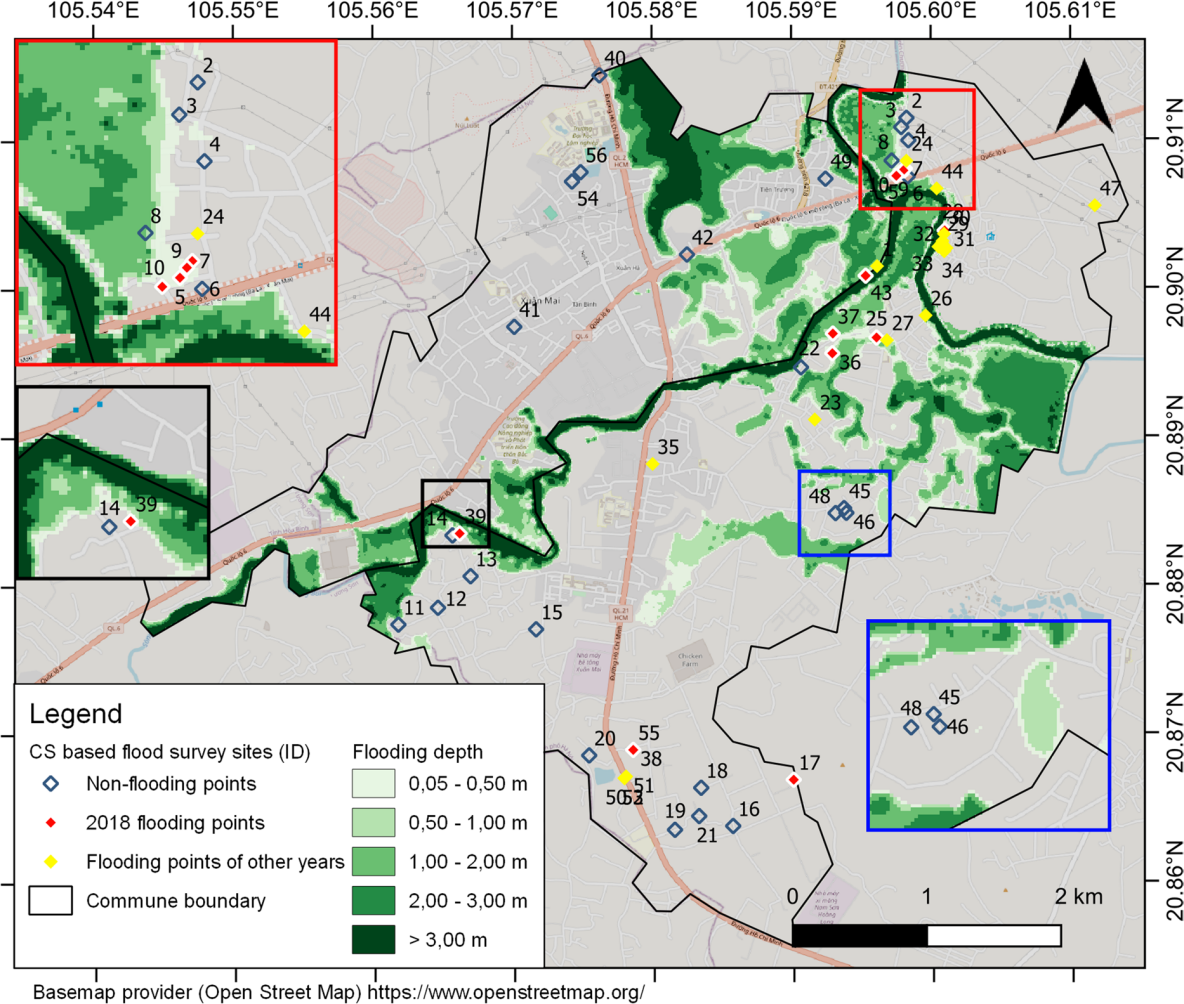
2018 flooding map of the study area and survey points from citizen scientists

There were 62 hazard data obtained from citizen scientists (Supplementary Material S5), of which 56 points lay inside the pilot area (Fig. 4). Of the 56 points, 25 had never been flooded and 31 were flooded in the past. Flood event chains such as 2013, 2017, 2018, 2019, and 2021 were frequently mentioned. Although the research focused on collecting flood events in the last 10 years, after 2013, the citizen science survey sites (ID) 47, 30, and 39 in Fig. 4 mentioned flood events in 2003 and 2008. In addition, the citizen science survey sites (ID: 7, 9, 10, 17, 39) included videos, photos, and additional information (failure of drainage systems, surveying locations compared with affected locations, etc.), which were used for the interpretation of citizen science data. The 2018 flood was the biggest in the last 10 years and was mentioned by 20 respondents. Twelve of 20 flooded points provided detailed information about the 2018 floods, such as flooding depth and duration, which were considered sufficient and consistent for this research. Therefore, we used 12 flooded points and 25 non-flooded points obtained from citizen scientists for comparison with a flooding map for 2018, as described in the next paragraph.

The 2018 flooding map was built using a topographic map and 89 flood depth points (Fig. 4). Once again, it is not guaranteed that this map represents the maximum flooding depth because it depends on the accuracy of measurements in residential areas (refer to the “Preliminary site visits” subsection). The map was used to compare with the flooding depth points obtained from citizen scientists. The 2018 flooding map was verified using statistical data. As the statistical data did not provide any information about the spatial distribution, the total estimated flooding area was compared with the statistically determined flooding area. The flooded area of the flooding maps was 447 ha, 2% higher than that of the statistical data, making it authoritative for comparison with citizen science data on flood hazard. The comparison results showed high overall agreement of 86% between citizen scientists’ flood survey points and the 2018 flooding map (Table 3). Non-flooded points gathered from citizen scientists were more reliable than flooded points; the agreement of these two classes was 96% and 67%, respectively. In addition, the flood depth of 8 flooded points gathered by citizen scientists was 0.34 m higher on average than the depth extracted from the flood map (Supplementary Material S5).

**Table 3:** Confusion matrix of flood hazard data of 2018 collected by citizen scientists and flooding map

Flooding map	Citizen scientists			
	Non-flooded points	Flooded points	Reference total	Participants’ agreement
Non-flooded points	24	4	25	96%
Flooded points	1	8	12	67%
			Total: 37	OA = 86%

#### Exposure data

The land use classification agreement level between citizen scientists and authors was assessed using a confusion matrix (Table 4). Eight citizen scientists were brought to 14 sites prepared by the authors to classify land use samples. One hundred land use samples, approximately 90% of the total expected samples (8 citizen scientists × 14 sites = 132 samples), were submitted by citizen scientists via digital data collection forms during the field experiment in the spring of April 2022 (Supplementary Material S6). The map of land use sample sites is shown in Fig. S3. The overall agreement was 0.82, which showed significant agreement in land use classification between citizen scientists and authors. Citizen scientists correctly classified high built-up, paddy rice, and water body areas without confusion. They also had almost perfect agreement in classifying forest and low built-up lands, with over 81% agreement for both classes. Non-rice and shrubland classes were the most confusing for participants, with only 47% of non-rice areas correctly classified by citizen scientists and 64% agreement for shrubland.

**Table 4:** Confusion matrix of land use classification by citizen scientists

Authors	Citizen scientists								
	High built-up	Low built-up	Rice	Non-rice	Water body	Forest	Shrubland	Reference total	Participants’ agreement
High built-up	16	0	0	0	0	0	0	16	100%
High built-up	2	11	0	0	0	0	0	13	85%
Rice	0	0	18	0	0	0	0	18	100%
Non-rice	0	3	6	7	0	0	0	15	47%
Water body	0	0	0	0	8	0	0	8	100%
Forest	0	0	0	0	0	13	3	16	81%
Shrubland	0	0	0	5	0	0	9	14	64%
								Total: 100	OA = 82%

#### Flood vulnerability

**Fig. 5:**
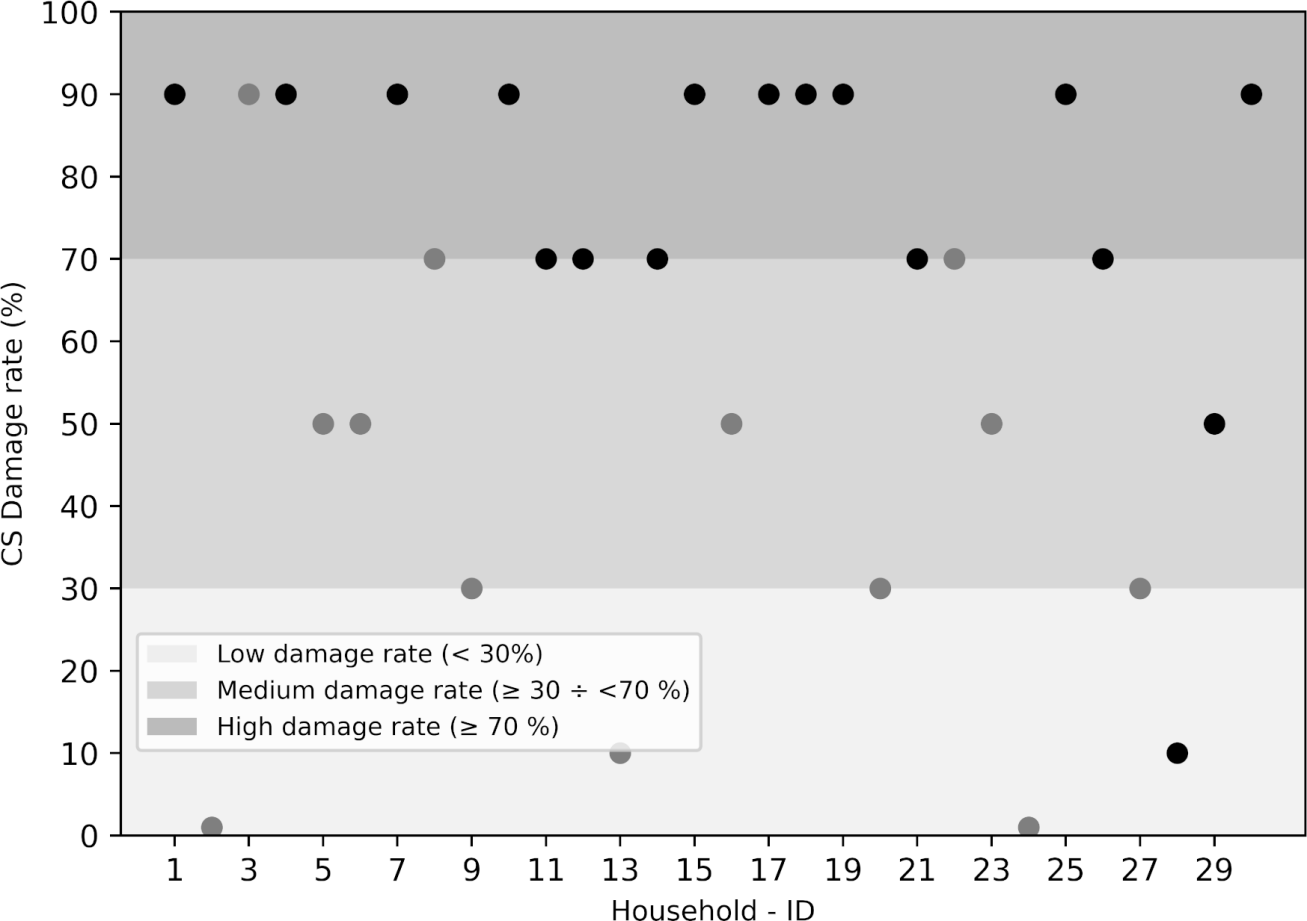
The paddy damage rate collected from citizen scientists for 30 households in the 2018 flood. Dots represent flood damage rates collected by citizen scientists; the black and gray colors in the dots represent households with damage levels ranging from 30 to 70% and over 70%, respectively, according to the local authority

Citizen scientists collected 46 flood vulnerability data with five households outside the pilot area, 11 not affected or having no names of households, and 30 households matching the list of flood-affected households on paddy fields recorded by the local authority after the 2018 flood (Supplementary Material S7). Therefore, the paddy damage data of these 30 households were compared to data from the local authority. A comparison of flood-affected households’ paddy damage rate between citizen scientists and the local authority is shown in Fig. 5 and Table 5. The overall agreement was 73%, demonstrating that citizen scientists have the potential to investigate or self-investigate data on flood vulnerability. The paddy damage rates collected from citizen scientists were lower than those collected from the local authority. For example, although all compared households had a damage rate greater than or equal to 30% according to the local authority, four households identified by citizen scientists had less than 30% damage or no damage (household IDs: 2, 13, 24, and 28, Fig. 5). Some of this disagreement might be affected by the time of data collection or by respondents’ memory or emotions.

**Table 5:** Confusion matrix of paddy damage rate data collected by citizen scientists

Local authority	Citizen scientists				
	Low damage	Medium damage	High damage	Reference total	Participants’ agreement
Low damage	0	0	0	0	-
Medium damage	3	7	3	13	54%
High damage	1	1	15	17	88%
				Total: 30	OA = 73%

### Monthly citizen science data collection

**Fig. 6:**
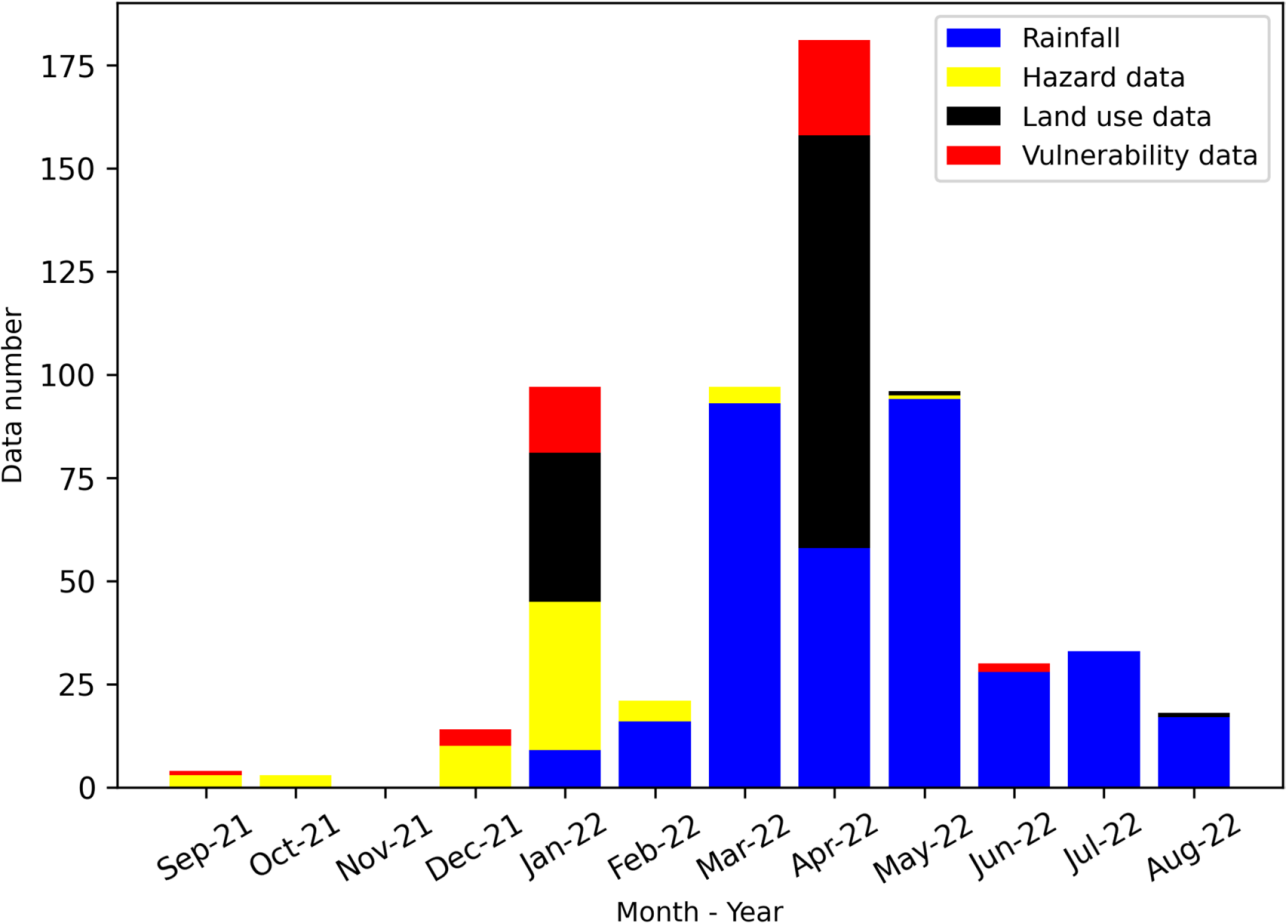
Monthly data contributed from citizen scientists for 1 year

**Fig. 7:**
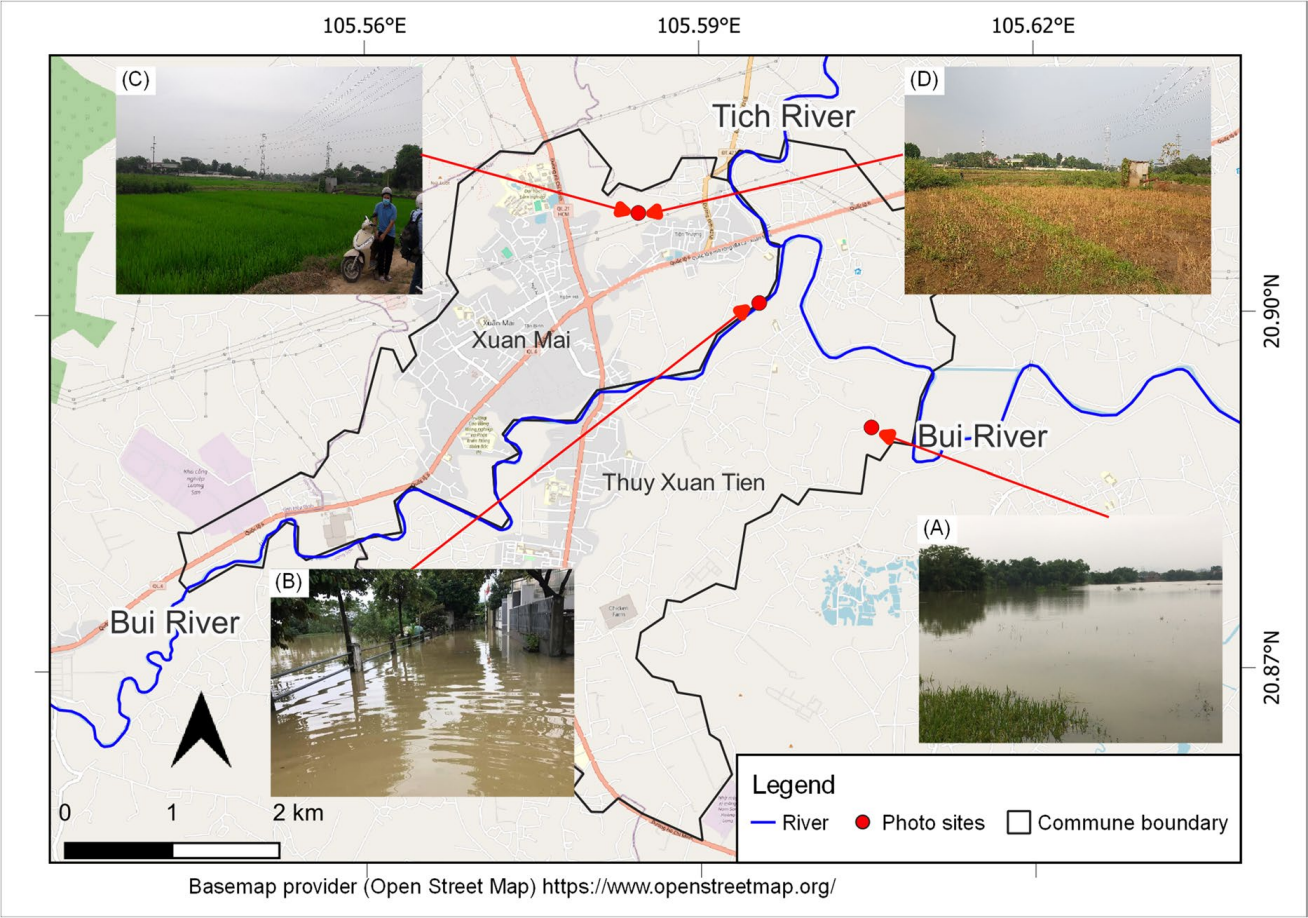
Location of citizen scientists reporting on flooding in residential area in October 2021 (A), flood damage to paddy fields in May 2022 (B), paddy-cultivated area in April 2022 (C), and paddy-abandoned area in August 2022 (D)

The monthly data gathered by participants from September 2021 to August 2022 are shown in Fig. 6 and Supplementary Material (S3, S4, S5, S6, and S7). In the first four months, the citizen science program was affected by the COVID pandemic, so this research obtained little data. After rainfall monitoring activities were implemented in January 2022, monthly data increased significantly in the first four months of 2022. April was the month with the largest quantity of data, with 181 data when a field experiment for land use collection took place. Monthly data gradually decreased in the last 3 months. During the data collection campaign, there was one moderate flood in October 2021 and an abnormally heavy storm in May 2022, which flooded residential areas and damaged paddies in the Bui River Basin, respectively. Citizen scientists living in flood-affected areas updated flood information in 2021 (Fig. 7A) and provided paddy damage after being harvested 1 month after the storm in 2022 (Fig. 7B). One citizen scientist gathered land use information in one area in April and August 2022, where a paddy-cultivated area was abandoned during flood reason in low-lying land (Fig. 7C and D).

## Discussion

### Citizen scientists’ engagement

Our results revealed that personal relationships strongly influenced citizen scientists’ recruitment. Nearly 50% of citizen scientists joined the citizen science program through introductions from friends and relatives, while 1% joined through social media. Vietnamese people are prone to collectivist norms (Ho et al., 2022). Hence, the participation of citizen scientists or the introduction of people with specific social standing in citizen science programs might affect the decisions of others. This finding is similar to the research results of community-based rainfall monitoring in Nepal by Davids et al. (2019). Unlike developing countries, in developed countries such as Germany and Italy, citizen science programs access citizens through unions, agencies, mass media (Pernat et al., 2021; Scaini et al., 2021; Schmitz et al., 2020), or individual invitations via email (Phillips et al., 2019). We assume that participants feel safe when they are informed about citizen science programs by friends and relatives. Our observations showed that some participants were concerned about the personal data protection and security of data collection applications (ODK Collect, web form).

Students are often efficient and primary contributors to citizen science programs (Davids et al., 2019; Prajapati et al., 2021). However, in predominantly rural study areas with low student density, recruiting student citizen scientists posed a challenge. According to Table 1 and Supplementary Material S3, which provides detailed information on participant demographics, only 40% of citizen scientists were students or postgraduates, and they were primarily recruited as investigators for flood surveying. Additionally, we attempted to involve students who have grown up in flood-affected areas to self-survey or measure rainfall during the weekend or social distancing periods due to the COVID pandemic. However, their participation was interrupted since they did not permanently reside in the study area. During the data collection campaign, we recruited school students and locals to participate in citizen science programs. Based on the data collected by the citizen scientists (refer to Table 2 and Supplementary Material S3 and S4), these participants tended to conduct simple measurements such as rainfall monitoring and land use sampling. In contrast, collecting flood hazard and vulnerability data requires evoking memory or utilizing several skills to exploit information from locals, particularly flood vulnerability information, which was often done by investigators.

Regular participation of citizen scientists is always a concern in citizen science programs. Similar to the research of Davids et al. (2019), citizen scientists recruited through social media and outreach methods actively participated in flooding surveys and measurements, surpassing those recruited through personal relationships. Although only one-fourth of the citizen scientists were recruited through the two above methods, they contributed nearly 50% of the total data (Supplementary Material S4). The results revealed that citizen scientists’ perceptions affected their active participation (Davids et al., 2019; Weeser et al., 2018). Therefore, outreach and social media are sustainable and promising methods to attract active participants. Although 50% of all citizen scientists participated in the project once, their single contributions were valuable for closing the information gap (Lowry et al., 2019), motivating other participants (Weeser et al., 2018), and cross-checking with other areas (Walker et al., 2016).

### Citizen science data quality

#### Flood hazard

Citizen scientists in flood-affected areas provided or gathered valuable insights about flooding. Locals in study areas often report more flood events than official reports (de Bruijn et al., 2019) and larger flood extents compared with focus group discussions (Canevari-Luzardo et al., 2017) or remote sensing image analysis (Sy et al., 2020). The overall agreement between plausible data from citizen science data and flooding maps was over 80% for floods in 2018, demonstrating citizen scientists’ potential for collecting and providing flood information regarding flooded and non-flooded points (Sy et al., 2020). Although some flooded surveying points are outside flooding maps, they provide valuable information for understanding flood impacts, as demonstrated by videos, photos, and detailed explanations provided by or gathered from citizen scientists (ID 17, Fig. 4). The flooding depth obtained from citizen scientists was higher than the flooding depth points extracted from the flooding map, which was also demonstrated in the results of Fohringer et al. (2015). These findings add to the evidence that information provided by the community is often overestimated compared to results measured by sensors and formal equipment (Fehri et al., 2020; Kipf et al., 2016).

#### Exposure data

This research revealed that citizen science is an appropriate way to collect land use information in the field as exposure data. The agreement between citizen scientists and authors in land use classification at 14 intended sites during the field experiment in April 2022 was high (OA > 80%). However, some aspects might have been affected by the results of land use sampling and classifying by citizen scientists. Ten percent of the expected samples were not submitted successfully, which might have been affected by data collection platform errors or participants who did not collect samples. Although the participants were asked to select surveying sites where they could determine a typical land use class within a 50-m diameter and take photos of 20 m × 20 m or 50 m × 50 m areas, some participants focused on particular objects (e.g., buildings, gardens, or rivers). These factors affected their misclassification when they did not consider the area proportion of the land use class. The participants faced challenges in classifying the land use class when it was located in a mixed area or included many different objects, which is consistent with the results of Sparks et al. (2015) and Visser (2015). However, the photos taken by participants could help users validate inappropriate information.

#### Flood vulnerability

There was decent agreement between the local authority and citizen science data on the paddy flood damage rate. The damage rates gathered by citizen scientists in this research were lower than those collected by the local government in 2018. The difference might have been affected by the memories of the respondents between the two surveys (Win et al., 2018). In addition, the respondents may have provided objective information when they felt recovered emotions from flood events (Glas et al., 2020). Furthermore, flood damage to paddy fields can be estimated more precisely when flood-affected paddy fields are harvested.

### Toward citizen-based flood monitoring and flood risk assessment

ICT development, especially mobile technology advances, is closely linked to citizen scientists’ engagement in documenting flood-related data during and after flood events (Fuchs et al., 2019). Citizen scientists can proactively share flood information through mobile data collection apps, including images, videos, and survey coordinates. This sharing enhances researchers’ understanding of flood impacts (Fohringer et al., 2015; Ribeiro et al., 2020). Community-based hydrologic monitoring networks serve as a two-way communication channel between locals, experts, and authorities (Ferri et al., 2020), promoting the data contribution of participants (Lowry et al., 2019) and updating flood situations, damage, and land use information. In this research, the rainfall monitoring network using low-cost rain gauges attracted locals’ attention to the citizen science program. Thanks to rainfall monitoring activities, citizen scientists can better understand the local rainfall information, and anticipate flooding potential, which can help participants take flood prevention measures (Ferri et al., 2020; Pandeya et al., 2021).

Citizens play a central role in modern flood risk assessment. Citizen engagement in flood risk assessment promotes the acceptance of flood control measures, thereby increasing their effectiveness (Cheung & Feldman, 2019). Citizen scientists can also contribute significantly to flood risk assessment by identifying hazardous areas along rivers (Scaini et al., 2021). Furthermore, Ferri et al. (2020) demonstrated that a citizen science approach to flood monitoring can reduce annual flood damages by nearly 50% by increasing community resistance and resilience. With the support of ICT, local people can contribute a wealth of information through websites and data collection applications to support flood risk assessment and management, especially in areas inaccessible to researchers or government agencies (Azizi et al., 2023; Ferri et al., 2020; Scaini et al., 2021).

### Research limitations

There are some limitations in the current study. First, the survey results might be influenced by the skills of the citizen scientists. Training sessions can boost participants’ confidence, and the survey results can be improved after each data collection (von Gönner et al., 2023). Second, this research only analyzed the implementation results for 1 year, revealing the potential of citizen science to fill the gap in historic flood data and update new flood events. Community-based flood data collection research is needed to establish partnerships with relevant agencies to determine common goals and work together effectively (Ferri et al., 2020). Sustainable citizen science projects require time and effort (Lowry et al., 2019), especially in developing countries such as Vietnam, where citizen science projects are limited (Tran et al., 2021a). Finally, although floods cause direct and indirect damage to many objects (e.g., buildings, infrastructures, life, crops), this study focused on collecting data on direct damage to rice. It is worth examining the potential of citizen scientists to collect direct and indirect damage data that depend on physical flood parameters such as flooding depth, duration, and velocity.

Some issues relating to the reference dataset need to be improved and addressed. The flooding map used for comparison with data from the citizen scientists depends on the accuracy of topographic maps, interpolation method applicability, and survey coordinates. To collect land use information in the field as exposure data, the research did not use corner coordinates of land use spots as in Visser’s approach Visser (2015) to reduce the complexity of the land use sampling procedures for the participants. This may lead participants to choose different land use classes at each site. In addition, reclassifying paddy damage rate levels from citizen scientists and local authorities into three levels may affect the analysis results. The paddy damage rate depends on multiple parameters, such as flooding depth, flood duration, and even seasonal variables, which can be used to evaluate the consistency of citizen science data in the future.

## Conclusions

This study demonstrated the applicability of citizen science in collecting flood risk-related data for 1 year for the Bui River Basin in Vietnam, focusing on flooding in residential area, land use information, and flood vulnerability to paddy fields. Citizen scientists who lived in or around flood-affected areas were recruited via various approaches to investigate or self-investigate flood risk-related data after participating in training sessions. Engaging citizen scientists in flood risk-related data collection generated plausible quality data and updated new flood situations by maintaining a community-based rainfall monitoring network. The following main conclusions are derived based on our analysis:

- Almost 50% of the participants were recruited through personal relatives. Nearly 50% of the completed questionnaires or measurements were reported by less than 10% of the participants. These “active participants” contributed over 50 data per person.
- Citizen science data can provide an additional source for flood risk assessment. The plausible data obtained from citizen scientists are consistent with the data from authors or the local authority and can be used for further studies. The videos, photos, and explanations collected from or by citizen scientists are crucial to understanding the investigated subjects.
- A community-based rainfall monitoring network using low-cost rain gauges revealed an effective communication channel to maintain the participants’ attention when providing past and present flood data.

We conclude that citizen scientists’ engagement in flood risk data collection not only fills the gap in flood data but also raises locals’ awareness of flood resilience building, which adheres to the Sendai framework for natural disaster reduction (United Nations Office for Disaster Risk Reduction, 2015). The citizen science approach is a good practice that can be applied to data-scarce regions or to collect other flood impacts to provide data to better understand flood impacts, assess flood risks, and propose appropriate solutions.

### Supplementary Information

Below is the link to the electronic supplementary material.

Supplementary file1 (DOCX 3709 KB)

Supplementary file2 (XLSX 78 KB)

## Data Availability

All data used in this study are included in the supplementary materials. The citizen science data collected through ODK Collect are available on the S4W data collection platform (https://data.smartphones4water.org/).
