# Supplementary material for: Citizen scientists’ engagement in flood risk-related data collection: a case study in Bui River Basin, Vietnam
Source: Environ Monit Assess. 2024 Feb 17;196(3):280. doi: 10.1007/s10661-024-12419-2 (PMC10874335; doi:10.1007/s10661-024-12419-2)
Supplement: Supplementary file 1 — Supplementary file1 (DOCX 3709 KB) [file 10661_2024_12419_MOESM1_ESM.docx]

Supplementary material for a publication in *Environmental Monitoring and Assessment*

**Citizen Scientists’ Engagement in Flood Risk-Related Data Collection: A case study in Bui River Basin, Vietnam**

Huan Ngoc Tran^*^, Martine Rutten, Rajaram Prajapati, Ha Thu Tran, Sudeep Duwal, Dung Trung Nguyen, Jeffrey Colin Davids, Konrad Miegel

* corresponding author

Email: huan.tran@uni-rosock.de

Affiliations: Faculty of Agricultural and Environmental Sciences, University of Rostock, Rostock, Germany

List of Contents

Supplementary material S1 Low-cost rain gauges

Supplementary material S2 Flood mark in the study area

Supplementary material S3 List of Participants (Excel file)

Supplementary material S4 List of flood data collection (Excel file)

Supplementary material S5 Hazard Data Collection and Analysis (Excel file)

Supplementary material S6 Exposure Collection (Map of land use sample sites and Excel file)

Supplementary material S7 Flood vulnerability Data Collection and Analysis (Excel file)

# S 1 – Low-cost rain gauges

The low-cost rain gauge was constructed using 1.5 to 2-liter soda bottles, concrete, and rulers, following a design inspired by David et al. (2019). Due to the rarity of uniform-diameter bottles in Vietnam, the majority of S4W rain gauges were fashioned from bottles with non-uniform diameters. Rainfall collected from these non-uniform diameter bottles was found to exceed that collected from bottles with uniform diameters by 5% to 20%. For a tutorial video demonstrating the construction of the S4W rain gauge in Vietnam, please refer to the following YouTube link (<https://bit.ly/3sciMeg>).


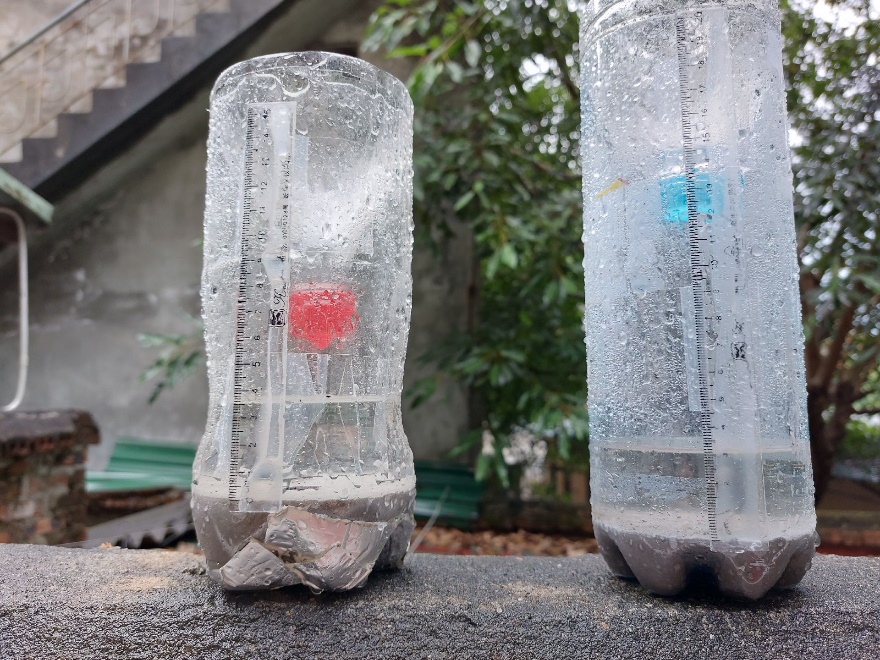


**Fig. S1** The rainfall in the ununiform and uniform diameter of the rain gauge were 38 and 32 mm, respectively on 12 May 2022 at Xuan Mai commune, Chuong My district, Hanoi city

# S 2- Flood mark in the study area


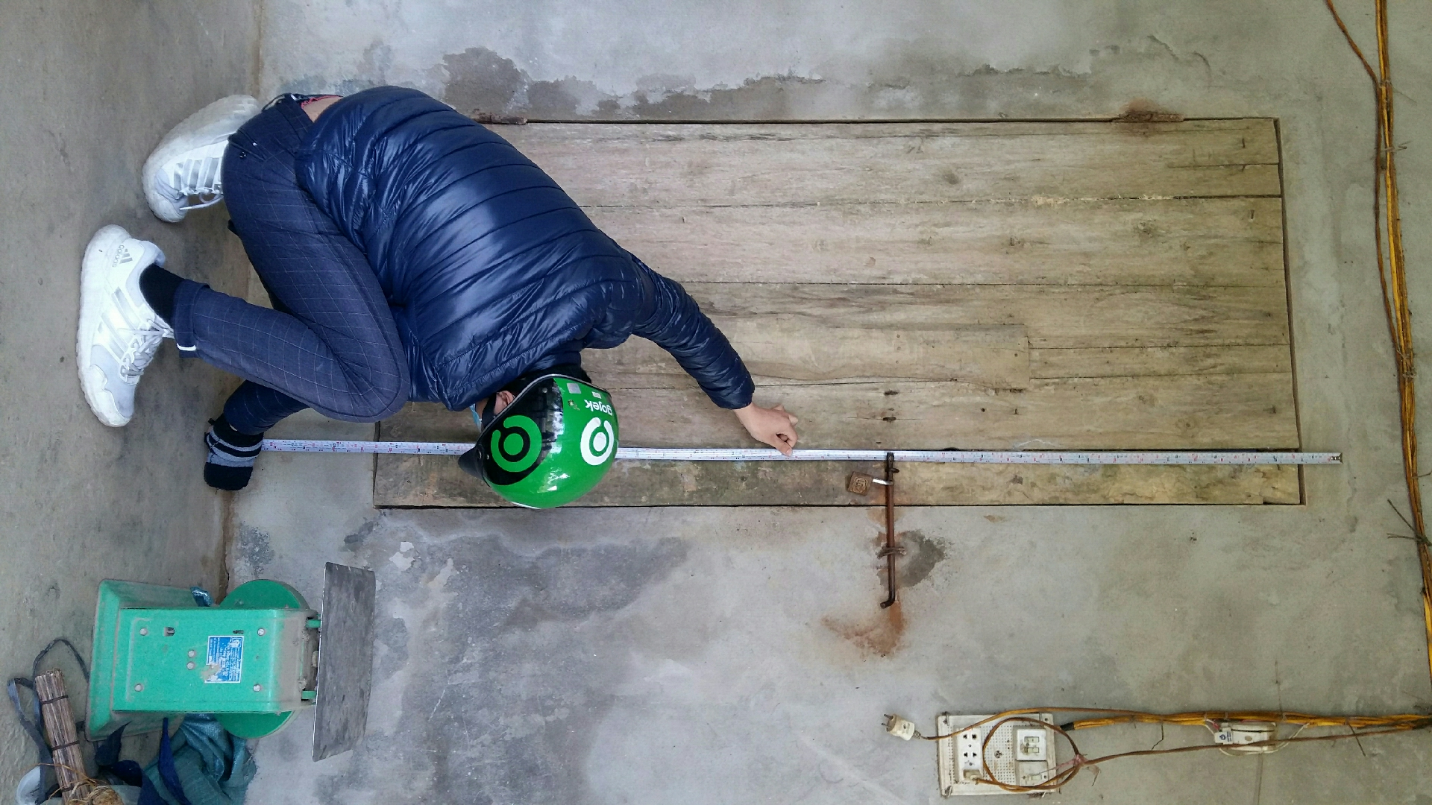


**Fig. S2** Flood mark at Xuan Linh hamlet, Thuy Xuan Tien commune, Chuong My, District, Hanoi in 2018 flood (Flooding depth: 2.5 m, Lat/ Long: 20.880, 105.562); Surveying date: Jan 04^th^, 2022) Link: <https://bit.ly/3qEQB7r>

# S 6 Exposure Collection


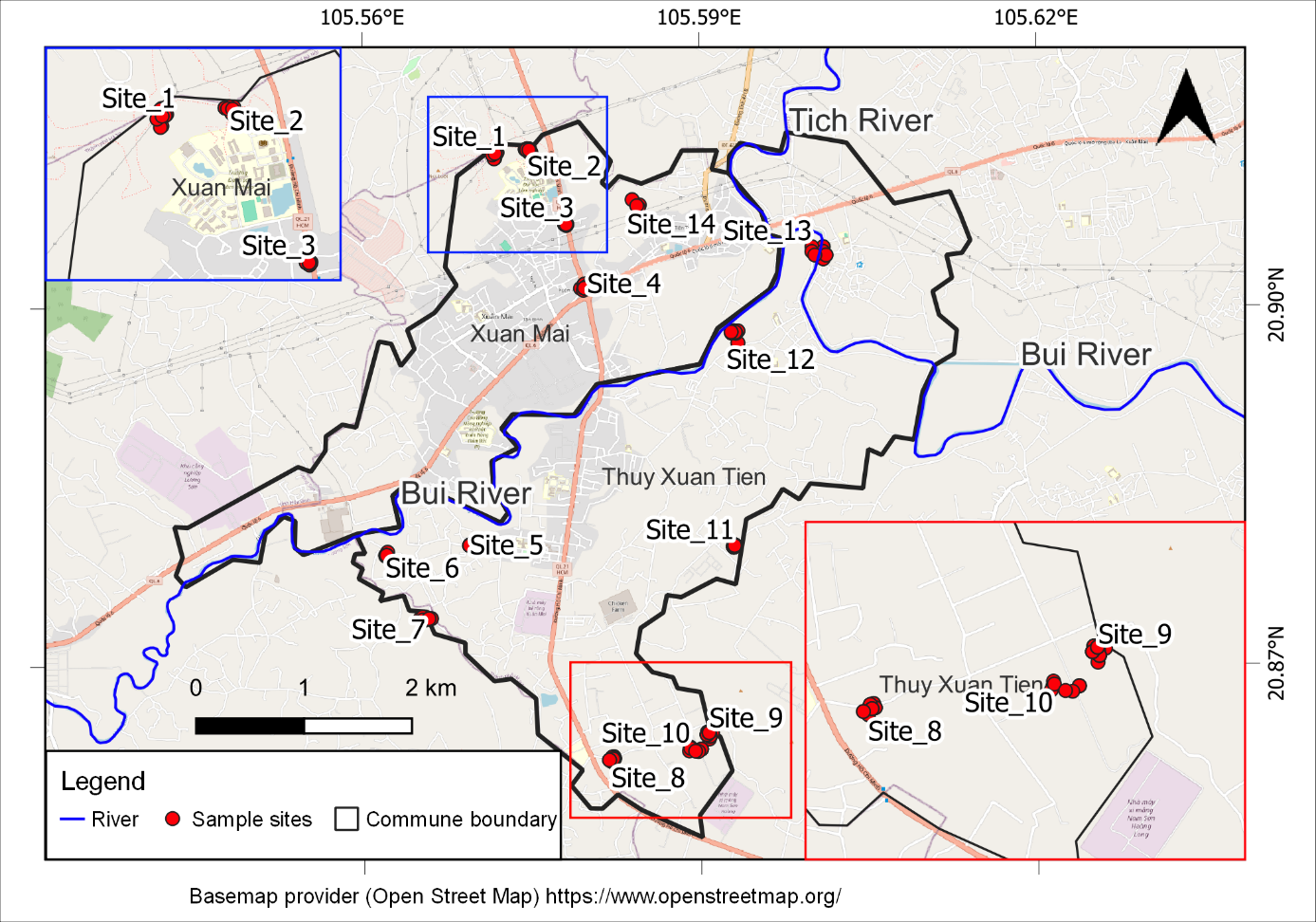


**Fig. S3** The map of land use sample sites
